# Supplementary material for: Spatial distribution of environmental DNA in a nearshore marine habitat
Source: PeerJ. 2017 Feb 28;5:e3044. doi: 10.7717/peerj.3044 (PMC5333549; doi:10.7717/peerj.3044)
Supplement: Supplemental Information 1 [file peerj-05-3044-s001.pdf]

Supplemental Material for

Spatial distribution of environmental DNA in a nearshore marine habitat

James L. O'Donnell*[1], Ryan P. Kelly[1], Andrew O. Shelton[2], Jameal F. Samhouri[3], Natalie C. Lowell[1,4], and Gregory D. Williams[5]

[1]School of Marine and Environmental Affairs, University of Washington, 3707 Brooklyn Ave NE, Seattle, Washington 98105, USA

[2]Earth Resource Technology, Inc., Under contract to the Northwest Fisheries Science Center, National Marine Fisheries Service, National Oceanic and Atmospheric Administration, 2725 Montlake Blvd E, Seattle, WA 98112, USA

[3]Conservation Biology Division, Northwest Fisheries Science Center, National Marine Fisheries Service, National Oceanic and Atmospheric Administration, 2725 Montlake Blvd E, Seattle, Washington 98112, USA

[4]School of Aquatic and Fishery Sciences, University of Washington, 1122 NE Boat St, Seattle, Washington 98105, USA

[5]Pacific States Marine Fisheries Commission, Under contract to the Northwest Fisheries Science Center, National Marine Fisheries Service, National Oceanic and Atmospheric Administration, 2725 Montlake Blvd E, Seattle, WA 98112, USA

January 19, 2017

*jodonnellbio@gmail.com

Methods

Bioinformatics

Reads passing the preliminary Illumina quality filter were demultiplexed on the basis of the adapter index sequence by the sequencing facility. We used fastqc to assess the fastq files output from the sequencer for low-quality indications of a problematic run. Forward and reverse reads were merged using PEAR v0.9.6 (Zhang et al., 2014) and discarded if more than 0.01 of the bases were uncalled. If a read contained two consecutive base calls with quality scores less than 15 (i.e. probability of incorrect base call = 0.0316), these bases and all subsequent bases were removed from the read. Paired reads for which the probability of matching by chance alone exceeded 0.01 were not assembled and omitted from the analysis. Assembled reads were discarded if assembled sequences were not between 50 and 168 bp long, or if reads did not overlap by at least 100 bp.

We used vsearch v2.1.1 (Rognes et al., 2016) to discard any merged reads for which the sum of the per-base error probabilities was greater than 0.5 ("expected errors") (Edgar, 2010). Sequences were demultiplexed on the basis of the primer index sequence at base positions 4-9 at both ends using the programming language AWK. Primer sequences were removed using cutadapt v1.7.1 (Martin, 2011), allowing for 2 mismatches in the primer sequence. Identical duplicate sequences were identified, counted, and removed in python to speed up subsequent steps by eliminating redundancy, and sequences occurring only once were removed. We checked for and removed any sequence likely to be a PCR artifact due to incomplete extension and subsequent mis-priming using a method described by Edgar (2010) and implemented in vsearch v2.1.1 (Rognes et al., 2016). Sequences were clustered into operational taxonomic units (OTUs) using the single-linkage clustering method implemented by swarm version 2.1.1 with a local clustering threshold (d) of 1 and fastidious processing (Mahé et al., 2014).

Cross-contamination of environmental, DNA, or PCR samples can result in erroneous inference about the presence of a given DNA sequence in a sample. However, other processes can contribute to the same signature of contamination. For example, errors during oligonucleotide synthesis or sequencing of the indexes could cause reads to be erroneously assigned to samples. The frequency of such errors can be estimated by counting the occurrence of sequences known to be absent from a given sample, and of reads that do not contain primer index sequences in the expected position

or combinations. These occurrences indicate an error in the preparation or sequencing procedures. We estimated a rate of incorrect sample assignment by calculating the maximum rate of occurrence of index sequences combinations we did not actually use, as well as the rates of cross-library contamination by counting occurrences of primer sequences from 12S amplicons prepared in a lab more than 1000 kilometers away, but pooled and sequenced alongside our samples. This represents a general minimum rate at which we can expect that sequences from one environmental sample could be erroneously assigned to another, and so we considered for further analysis only those reads occurring with greater frequency than this across the entire dataset.

We checked for experimental error by evaluating the Bray-Curtis similarity (1 - Bray-Curtis dissimilarity) among replicate PCRs from the same DNA sample. We calculated the mean and standard deviation across the dataset, and excluded any PCR replicates for which the similarity between itself and the other replicates was less than 1.5 standard deviations from the mean.

To account for variation in the number of sequencing reads (sequencing depth) recovered per sample, we rarefied the within-sample abundance of each OTU by the minimum sequencing depth (Oksanen et al., 2016).

Because each step in this workflow is sensitive to contamination, it is possible that some sequences are not truly derived from the environmental sample, and instead represent contamination during field sampling, filtration, DNA extraction, PCR, fragment size selection, quantitation, sequencing adapter ligation, or the sequencing process itself. We take the view that contaminants are unlikely to manifest as sequences in the final dataset in consistent abundance across replicates; indeed, our data show that the process from PCR onward is remarkably consistent. Thus, after scaling to correct for sequencing depth variation, we calculated from our data the maximum number of sequence counts for which there is turnover in presence-absence among PCR replicates within an environmental sample. We use this number to determine a conservative minimum threshold above which we can be confident that counts are consistent among replicates and not of spurious origin, and exclude from further analysis observations where the mean abundance across PCR replicates within samples does not reach this threshold. For further analyses we use the mean abundance across PCR replicates for each of the 24 environmental samples.

In order to determine the most likely taxon from which each sequence originated, the representative sequence from each OTU was then queried against the NCBI nucleotide collection (GenBank;

version October 7, 2015; 32,827,936 sequences) using the blastn command line utility (Camacho et al., 2009). In order to maximize the accuracy of this computationally intensive step, we implemented a nested approach whereby each sequence was first queried using strict parameters (e-value = 5e-52), and if no match was found, the query was repeated with decreasingly strict e-values (5e-48 5e-44 5e-40 5e-36 5e-33 5e-29 5e-25 5e-21 5e-17 5e-13). Other parameters were unchanged among repetitions (word size: 7; maximum matches: 1000; culling limit: 100; minimum percent identity: 0). Each query sequence can be an equally good match to multiple taxa either because of invariability among taxa or errors in the database (e.g. human sequences are commonly attributed to other organisms when they in fact represent lab contamination). In order to guard against these spurious results, we used an algorithm to find the lowest common taxon for at least 80% of the matched taxa, implemented in the R package taxize 0.7.8 (Chamberlain and Szöcs, 2013; Chamberlain et al., 2016). Similarly, we repeated analyses using the dataset consolidated at the same taxonomic rank across all queries, for the rank of both family and order.

Alternative distance decay model formulations

Linear: We fit a straight line through the points after log-transforming the spatial distances to estimate the intercept and slope. This model ignores the bounds of our response variable of community similarity.

Michaelis-Menten: We fit a Michaelis-Menten-like curve to our data. Our formulation can be thought of as a modification of the Michaelis-Menten equation, but with the addition of a parameter in the numerator which modifies the intercept.

$$y = \frac{AB + Cx}{B + x} \tag{1}$$

Where C is the asymptote of minimum similarity. This formulation allows us to estimate the maximum similarity in the system, and the rate at which it is achieved. If the value of the parameter (AB) is 0 (i.e. if the intercept is 0), the form is identical to the Michaelis-Menten equation:

$$y = \frac{Cx}{B + x} \tag{2}$$

This is conceptually satisfying in that a fit through [0,1] reflects the theoretical expectation that samples at zero distance from one another are necessarily identical. Given an efficient sampling technique, replicate samples taken at the same position in space should be identical, and thus the intercept of the regression of similarity against distance should be 1, and deviation from 1 is an indicator of the efficiency of the sampling method.

Finally, we considered a model which estimates an asymptote as the total change in similarity (D):

$$y = \frac{A + Dx}{B + x} \tag{3}$$

However, this model failed to converge and produced uninformative estimates of all parameters.

References

Camacho, C., Coulouris, G., Avagyan, V., Ma, N., Papadopoulos, J., Bealer, K., and Madden, T. L. (2009). BLAST+: architecture and applications. *BMC Bioinformatics*, 10:421.

Chamberlain, S. A. and Szöcs, E. (2013). taxize: taxonomic search and retrieval in R. *F1000Research*, 2(0):191.

Chamberlain, S. A., Szöcs, E., Boettiger, C., Ram, K., Bartomeus, I., Foster, Z., and O'Donnell, J. L. (2016). taxize: Taxonomic information from around the web. R package.

Edgar, R. C. (2010). Search and clustering orders of magnitude faster than BLAST. *Bioinformatics*, 26(19):2460–2461.

Mahé, F., Rognes, T., Quince, C., de Vargas, C., and Dunthorn, M. (2014). Swarm: robust and fast clustering method for amplicon-based studies. *PeerJ*, 2:e593.

Martin, M. (2011). Cutadapt removes adapter sequences from high-throughput sequencing reads. *EMBnet.journal*, 17(1):10.

Oksanen, J., Blanchet, F. G., Friendly, M., Kindt, R., Legendre, P., McGlinn, D., Minchin, P. R., O'Hara, R. B., Simpson, G. L., Solymos, P., Stevens, M. H. H., Szoecs, E., and Wagner, H. (2016). vegan: Community Ecology Package.

Rognes, T., Flouri, T., Nichols, B., Quince, C., and Mahé, F. (2016). VSEARCH: a versatile open source tool for metagenomics. *PeerJ*, 4:e2584.

Zhang, J., Kobert, K., Flouri, T., and Stamatakis, A. (2014). PEAR: A fast and accurate Illumina Paired-End reAd mergeR. *Bioinformatics*, 30(5):614–620.

Supplemental Figures

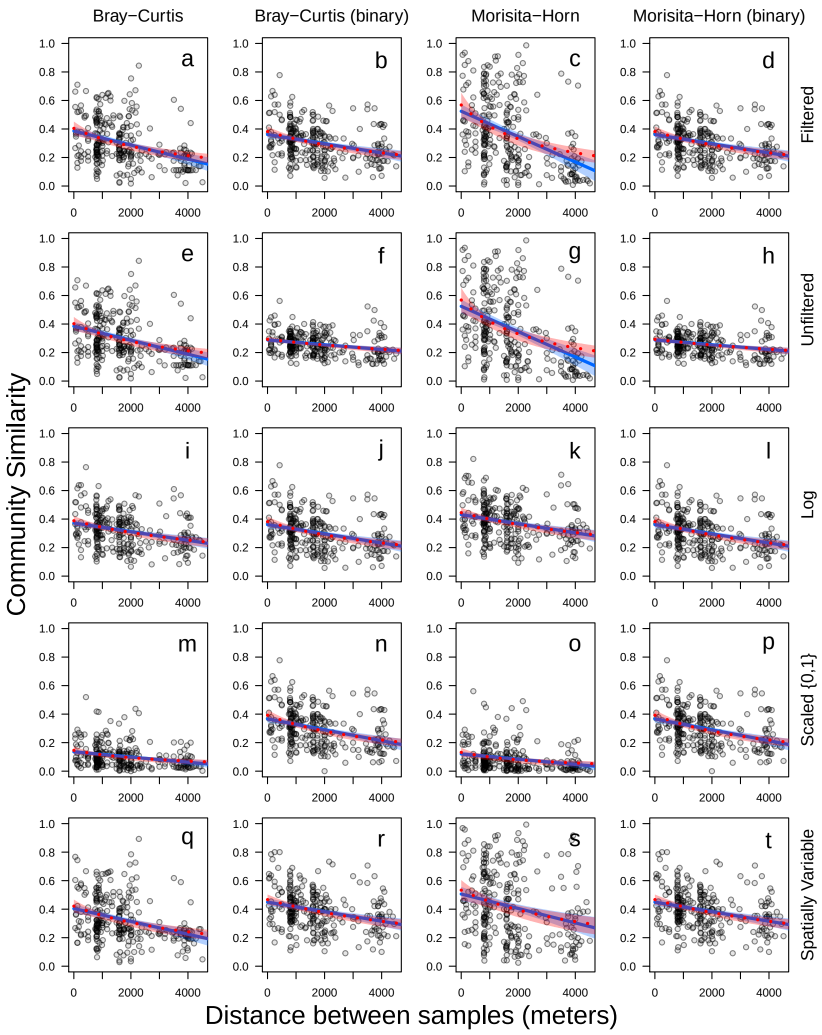

Figure 1: Distance decay relationship of environmental DNA communities using a variety of models, metrics, and data subsets. Each point represents the similarity of a site sampled along three parallel transects comprising a 3000 by 4000 meter grid. Each row of plots represents a different data subset indicated in the right margin, including the final filtered data reported in the main text (a-d), the unfiltered data including all rare OTUs (e-h), log-transformed (log(x+1)) data (i-l), OTU abundance scaled relative to within-taxon maximum (m-p), and exclusion of OTUs found at only one site (q-t). Columns indicate the similarity index used (Bray Curtis or Morisita-Horn) and whether the input was full abundance data or binary (0,1) transformed data. Lines and bands illustrate the fit and 95% confidence interval of both the main nonlinear model (red, dashed line) and a simple linear model (blue, solid line). Results using the Jaccard distance are omitted because of its similarity to Bray-Curtis.

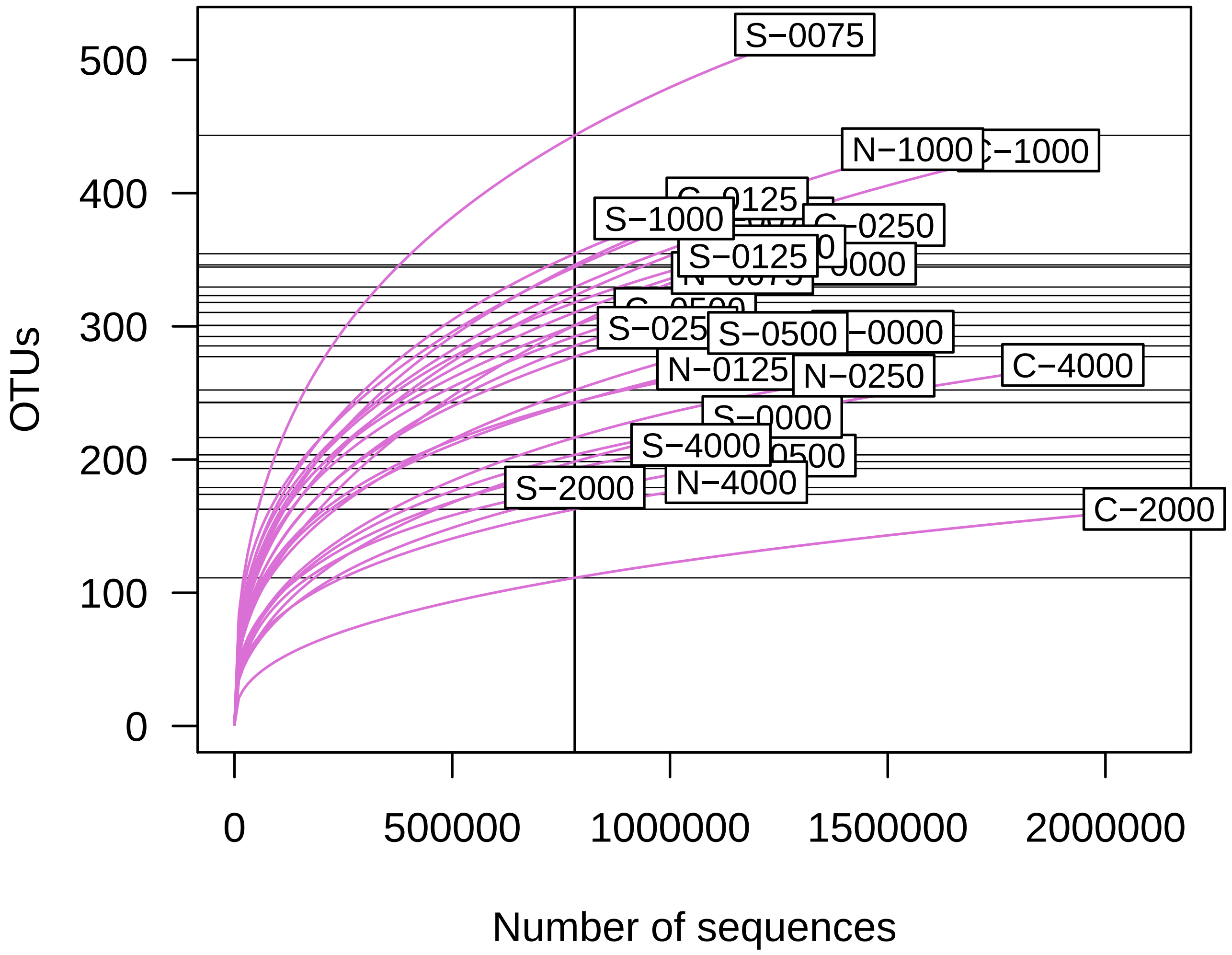

Figure 2: Accumulation of OTUs from 24 environmental samples using randomized rarefaction. Four replicate PCRs were conducted using DNA each environmental sample and independently sequenced, but these are collapsed here to illustrate a single representation of richness. Sample names indicate the position in the sampling grid: south (S), central (C), or north (N), followed by the distance along the transect, in meters (0, 75, 125, 250, 500, 1000, 2000, 4000). Vertical line indicates the minimum combined number of sequence reads per sample. Horizontal lines indicate OTU richness for each sample at the minimum combined number of sequence reads.

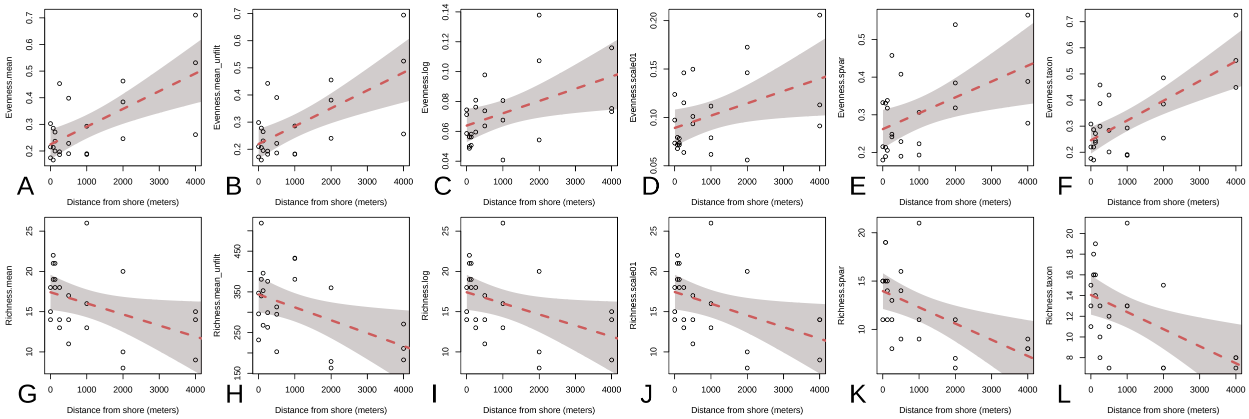

Figure 3: Aggregate diversity metrics of each site plotted against distance from shore. Both Simpson's Index (top; A-F) and richness (bottom; G-L) are shown for a variety of data subsets and transformations (left to right: mean (A,G), unfiltered mean (B,H), log(x + 1) transformed (C,I), scaled (D,J), spatially variable (E,K), and taxon clustered (F,L)). Lines and bands illustrate the fit and 95% confidence interval of a linear model. See methods text for detailed data descriptions.
